# Laparoscopic resection of hepatic epithelioid hemangioendothelioma: report of eleven rare cases and literature review

**DOI:** 10.1186/s12957-020-02034-z

**Published:** 2020-10-29

**Authors:** Jianjun Xu, Shaobo Hu, Suzhen Li, Weimin Wang, Xing Zhou, Yuzhe Wu, Zhe Su, Xiang Cheng, Yang Gao, Qichang Zheng

**Affiliations:** 1 Department of Hepatobiliary Surgery, Union Hospital, Tongji Medical College, Huazhong University of Science and Technology, Wuhan, 430022 China; 2 Department of General Surgery, The People’s Hospital of Honghu City, Honghu, 433200 China; 3 Department of Gastroenterology, Wuhan Asia General Hospital, Wuhan, 430056 China

**Keywords:** Hepatic epithelioid hemangioendothelioma, Liver neoplasms, Laparoscopic hepatectomy, Targeted therapy

## Abstract

**Background:**

Hepatic epithelioid hemangioendothelioma (HEHE) is an extremely rare borderline tumor of vascular endothelial origin. Laparoscopic resection of HEHE has never been reported.

**Methods:**

The clinical data of eleven patients with HEHE (4 women and 7 men) who were diagnosed and treated at the Union Hospital (Wuhan, China), and Wuhan Asia General Hospital (Wuhan, China), between March 2012 and July 2020 were analyzed retrospectively.

**Results:**

The mean age of HEHE patients was 42.4 ± 13.9 years (range 22–67 years). All patients underwent laparoscopic surgery alone or in combination with radiofrequency ablation. Most tumors showed aggressive growth or metastasis. By immunohistochemistry, tumor cells were positive for CD31, CD34, ERG, PCK, FLi-1, TFE-3, and Ki-67 (labeling index range, 5–15%). In one of the patients, the tumor was accompanied by partial necrosis with a local appearance of epithelioid angiosarcoma. Postoperative adjuvant treatment included chemotherapy, sorafenib, and Huaier granule. As of July 2020, the median follow-up duration was 36 months (range, 9–60 months), with 2 (18.2%) patients experiencing tumor recurrence.

**Conclusions:**

This is the first report of laparoscopic hepatectomy of HEHE. Curative laparoscopic hepatectomy might be an acceptable treatment for appropriate HEHE patients.

## Introduction

Tumors of the liver are common, but the primary or secondary hepatic epithelioid hemangioendothelioma (HEHE) is rare [1]. In the majority of HEHE patients, multiple nodules are secondary and result from a systemic spreading of the disease. Epithelioid hemangioendothelioma occurs most frequently in the liver [2], lung [3], and bone [4]. HEHEs are extremely rare borderline tumor, while primary single nodule HEHE is even rarer due to its high metastatic potential. The definitive diagnosis of HEHE requires histopathological evaluation. At present, there is no consensus on the optimal treatment for HEHE, but surgical excision is often considered an effective treatment [5]. However, laparoscopic resection of HEHE is seldom reported in the literature. This report describes cases of curative laparoscopic resection of primary HEHE accompanied by vascular invasion.

## Patients and methods

This retrospective study included eleven HEHE patients diagnosed on the basis of postoperative histopathology. These patients were treated between March 2012 and July 2020 at the Union Hospital, Tongji Medical College, Huazhong University of Science and Technology (Wuhan, China), and Wuhan Asia General Hospital (Wuhan, China). The following data were extracted from the medical records of the patients: age, gender, results of physical examination, blood test results, treatment protocol, histopathology findings, and follow-up data.

The patients were evaluated at the hospital at 1 month after the operation, and then every 3 months for the next 2 years. Subsequently, they were examined every 6 months until the last follow-up (July 2020). Standard physical examination, blood test, and imaging were performed at every review. The final status of the patients was classified as tumor-free survival, tumor recurrence, or death. Tumor-free survival was calculated from the time of HEHE diagnosis to the time of recurrence.

This research was approved by the Ethical Review Committee of the Tongji Medical College, and informed consent was obtained from the study participants.

## Results

Eleven patients, seven males and four females, diagnosed with HEHE were included in this retrospective study. Their basic clinical data are listed in Table 1. The mean age of patients was 42.4 ± 13.9 years (range 22–67 years). Clinical symptoms included right upper abdominal pain, abdominal distension, jaundice, and fever. Biochemical test documented an increase in alkaline phosphatase, γ-glutamyl transferase, and lactate dehydrogenase. The number of tumors varied from 1 to 3, and all the lesions were located in the same hepatic lobe. Their diameter ranged from 1 to 9 cm. All patients underwent CT and MRI scans. The enhanced CT and MRI imaging demonstrated the absence of intratumoral enhancement, but an evident enhancement of liver parenchyma at the edge of the lesion. Prior to operation, patient 9 and 10 received an ultrasound-guided liver biopsy of the liver lesion, which showed a large amount of hepatocellular necrosis mixed with some epithelial cells and failed to confirm the diagnosis of HEHE. All eleven patients underwent laparoscopic resection during which radiofrequency ablation (RFA) was performed on the surgical margin in some patients. The pathologic examination confirmed that all eleven patients underwent laparoscopic regular hepatic lobectomy with R0 resection. The surgical margin was more than 2 cm from the lesion. Postoperative adjuvant treatment included chemotherapy, sorafenib, and Huaier granule [6].

**Table 1:** Clinical data of hepatic epithelioid hemangioendothelioma patients

Patient	Age	Gender	Follow-up duration (months)	Number of tumor	Metastasis	Pathology	Treatment	Recurrence
1	35	Male	60	1	No	CD31(+), CD34(+), ERG(+), TFE-3(+), Ki67 (LI: approximately 5%)	LH	No
2	43	Male	48	2	Lymph nodes	CD31(+), CD34(+), ERG(+),PCK(+), Ki67 (LI: approximately 10%)	LH + RFA + lymphadenectomy	Yes
3	67	Female	48	1	No	CD31(+), CD34(+), ERG(+), TFE-3(+), Ki67 (LI: approximately 5%)	LH	No
4	41	Male	42	1	No		LH + RFA	No
5	28	Female	42	2	Lymph nodes	CD31(+), CD34(+), ERG(+), FLi-1(+), Ki67 (LI: <5%)	LH + lymphadenectomy	Yes
6	22	Male	36	1	No	CD31(+), CD34(+), ERG(+), Ki67 (LI: approximately 5%)	LH + Huaier granule	No
7	53	Male	36	2	No	CD31(+), CD34(+), ERG(+), PCK(+), Ki67 (LI: approximately 5%)	LH + sorafenib + Huaier granule	No
8	39	Female	24	1	No	CD31(+), CD34(+), ERG(+), FLi-1(+), Ki67 (LI: <5%)	LH	No
9	28	Male	12	3	Gallbladder	CD31(+), CD34(+), ERG(+), PCK(+), Ki67 (LI: approximately 5%)	LH + RFA + cholecystectomy + chemotherapy + sorafenib + Huaier granule	No
10	55	Female	12	2	Lymph nodes	CD31(+), CD34(+), ERG(+), Ki67 (LI: approximately 15%)	LH + RFA + lymphadenectomy + sorafenib + Huaier granule	No
11	55	Male	9	1	No	CD31(+), CD34(+), ERG(+), TFE-3(+), Ki67 (LI: approximately 5%)	LH + sorafenib + Huaier granule	No

*LH* laparoscopic hepatectomy, *RFA* radiofrequency ablation

The histopathological findings documented that most of cross-section of the tumor were hoary. In some patients, tumor tissue exhibited necrosis and calcification. Especially in patient 10, the HEHE was accompanied by partial necrosis with a local appearance of epithelioid angiosarcoma. Immunohistochemical staining indicated that tumor cells were positive for CD31, CD34, ERG, PCK, FLi-1, TFE-3, and Ki67 (labeling index, 5–15%).

As of July 2020, the median duration of the follow-up was 36 months (range, 9–60 months), and all patients survived with 2 (18.2%) patients experiencing local recurrence in the liver. None of the patients had postoperative distant metastasis. In addition, both patients with recurrence underwent CT-guided radiofrequency ablation of the percutaneous liver and achieved a radical curative effect.

### Presentation of a typical case

**Fig. 1:**
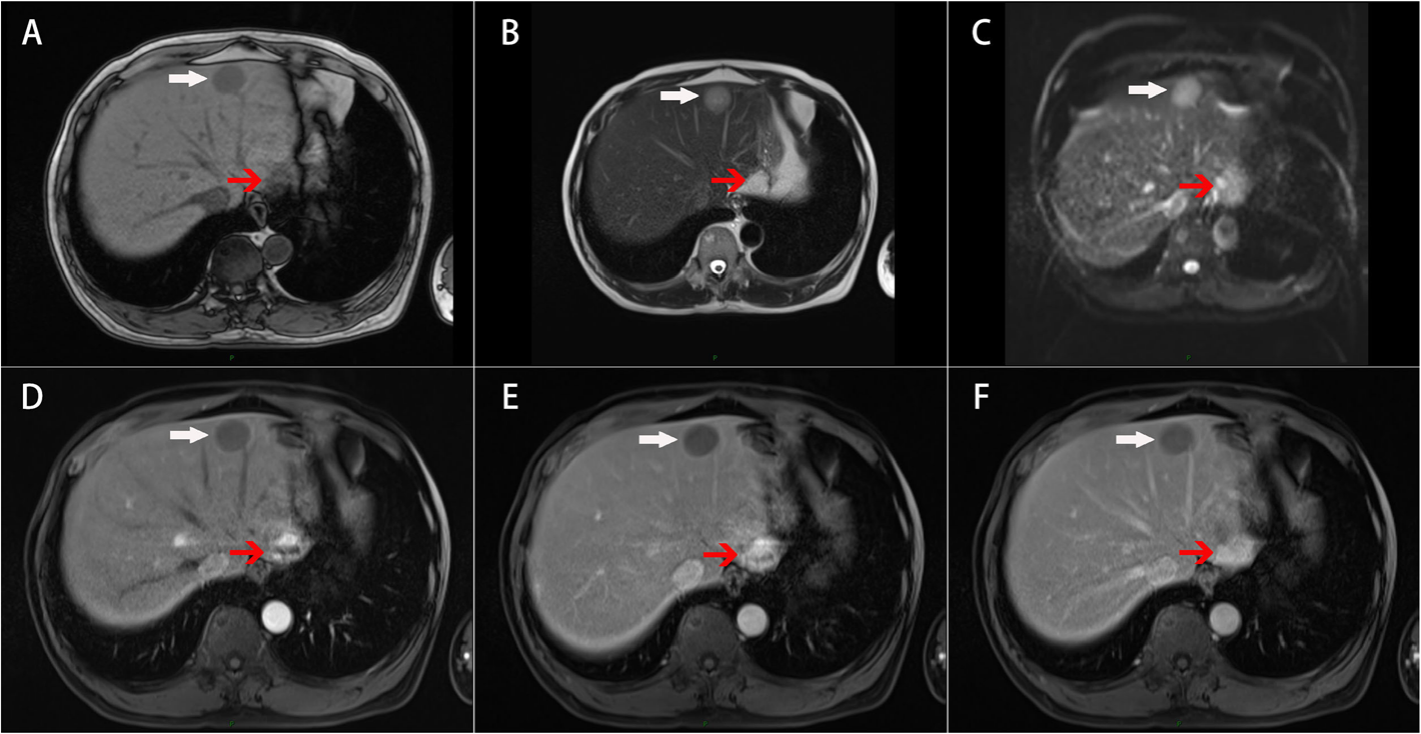
Enhanced MRI scans. **a** Hypointense on T1-weighted images (white arrow). **b** Hyperintense on T2-weighted images. **c** DWI showed mild limitation of dispersion. **d** Ring-shaped enhancement. **e** Portal vein stage, slight ring-shaped enhancement. **f** Delayed stage, decreased enhancement. Cavernous hemangioma (red arrow)

**Fig. 2:**
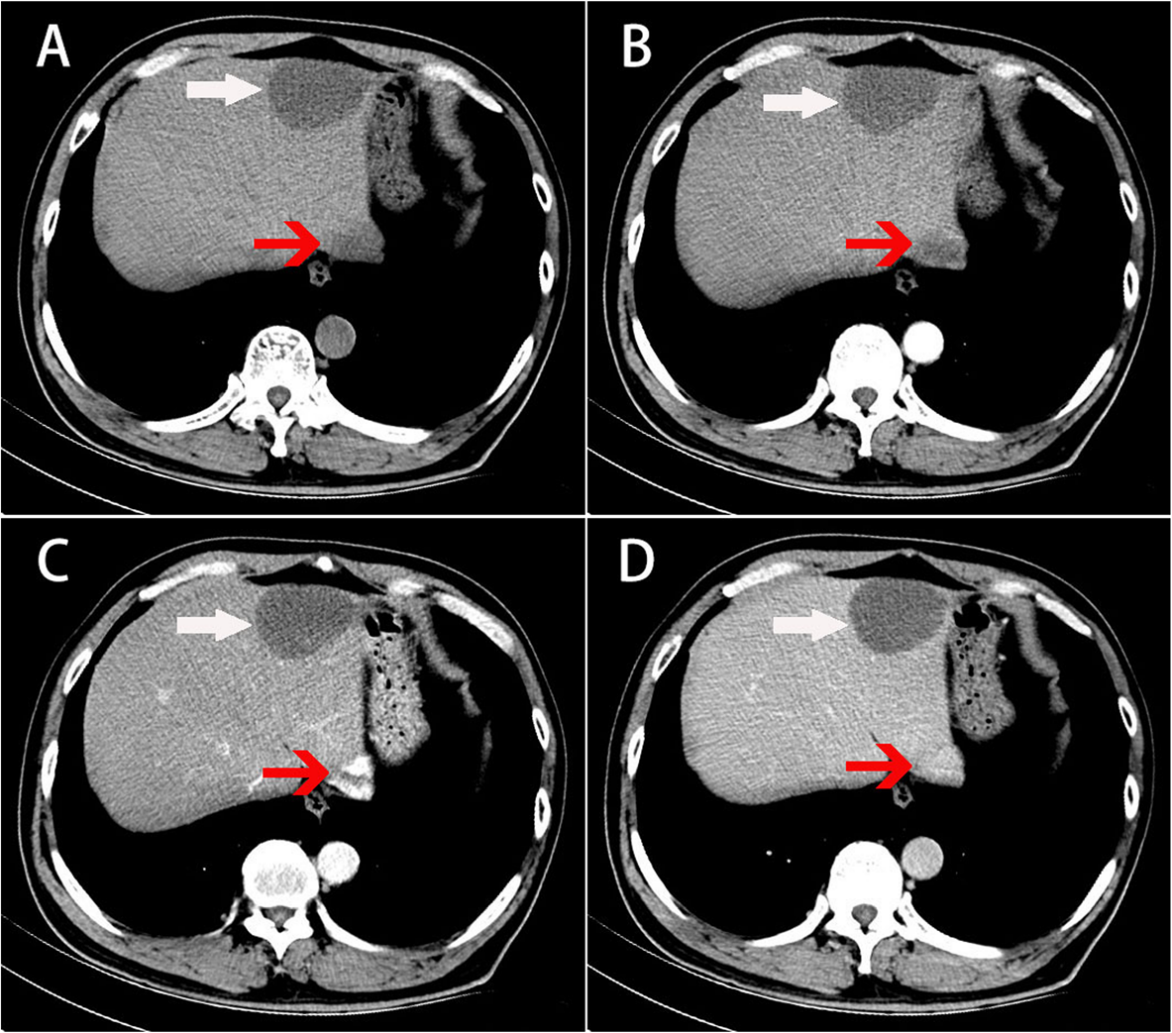
Enhanced CT scans. **a** Plain scan, **b** arterial stage, **c** portal vein stage, **d** and delayed stage. A patchy low-density shadow without significant enhancement can be seen in the liver (white arrow). Cavernous hemangioma (red arrow)

A 55-year-old male patient had a progressively enlarging mass in the liver for one year. No abnormalities were found in routine blood tests. The coagulation function was normal. Biochemical tests demonstrated that the levels of indicators of liver and kidney function were also in the normal range. Additionally, tumor markers were not elevated. MRI imaging revealed that the subcapsular lesion of the SII segment of the liver was hypointense on T1-weighted images; the size of the lesion was approximately 2.6 × 1.8 cm, and its boundary was clearly defined (Fig. 1). The lesion was slightly hyperintense on T2-weighted images. Diffusion-weighted imaging (DWI) demonstrated a mild limitation of dispersion. Enhanced MRI showed a slight ring-shaped enhancement after the administration of contrast agent. Additionally, enhanced MRI documented the presence of several typical cavernous hemangiomas in the liver; the largest of them was approximately 2.7 × 2.2 cm and was located in the SII segment of the liver (red arrow). At the time of imaging, the liver mass was considered to be benign, and a follow-up was recommended. The second imaging of the liver mass was performed by computed tomography (CT) after 1 year of follow-up (Fig. 2). CT scans revealed a patchy low-density shadow (white arrow), approximately 4.5 × 5.8 cm in size, under the capsule of the SII segment of the liver. The subcapsular lesion of the SII segment of the liver did not exhibit significant enhancement. Numerous typical cavernous hemangiomas (red arrows) with progressive enhancement on the enhanced CT scan were found in the liver; the largest of them, approximately 2.7 × 2.2 cm, was located in the SII segment of the liver. A number of small cysts were also detected in the liver, the larger of them reaching about 2–3 mm in diameter. Preoperatively, the patient differential diagnosis was “1. malignant tumor of the liver; 2. cavernous hemangioma of the liver; 3. hepatic cyst”. Taking into consideration the possibility of biopsy failure, bleeding, tumor metastasis, and the feasibility of complete resection of the liver mass, the decision was made to conduct laparoscopic hepatectomy of the left lateral hepatic lobe without performing a preoperative biopsy. After the procedure, the patient received sorafenib and Huaier granule as adjuvant therapy [6]. The patient was discharged 5 days after laparoscopic surgery. A follow-up at 9 months did not detect the recurrence of the tumor, and curative resection was asserted.

### Pathology

**Fig. 3.**
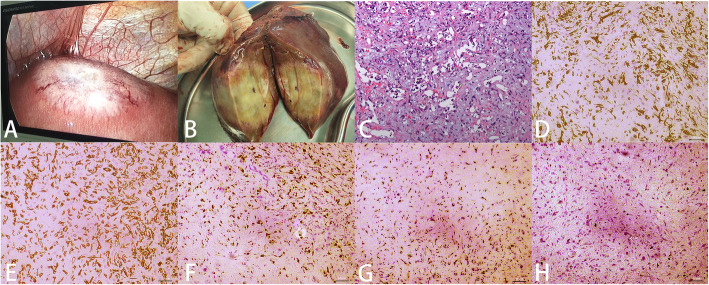
Pathological examination. **a** Laparoscopic view, **b** postoperative gross solid horay specimens, and **c** histopathological section confirmed hepatic epithelioid hemangioendothelioma (HE, × 200). Immunohistochemical staining documented the tumor was positive for CD31 (**d**), CD34 (**e**), ERG (**f**), and TFE-3 (**g**). Ki67 was approximately 5%

The lesion was located under the capsule of the left lateral lobe of the liver (Fig. 3a). Its surface was slightly concave, a large number of new blood vessels were noted, and peritoneal adhesions were observed around the liver. The lesion was firm, and the sections were hoary (Fig. 3b). No tumor involvement was found at the surgical margin, indicating R0 resection. A definitive diagnosis of epithelioid hemangioendothelioma with microvascular invasion was confirmed by postoperative pathological examination (Fig. 3c). By immunohistochemistry, the tumor was positive for CD31, CD34, ERG, and TFE-3 and negative for PCK, CK8/18, EMA, CK19, Glypican3, hepatocytes, and arginase. Approximately 5% of tumor cells were Ki67-positive.

### Literature review

Any searchable literature in the PubMed, Google Scholar, and Web of Science databases concerning surgical treatment of primary HEHE, whatever language it was published in, is included. The search term used was (((((Epithelioid Hemangioendothelioma) OR (Epithelioid Hemangioendotheliomas)) OR (Hemangioendotheliomas, Epithelioid) AND ((liver) OR (hepatic) AND)) AND (((surgery) OR (operati*)) OR (therapy)) AND (surviv*). A total of 44 papers were retrieved. Finally, twenty papers on surgical treatment of HEHE and providing survival information were listed in Table 2. The references mentioned in Table 2 are included in the supplementary document.

**Table 2:** Summary of operative treatment of outcomes of hepatic epithelioid hemangioendothelioma with survival data in reviewed literatures

Author (year)	No.	Diagnostic methods	Tumor size, mean, cm	Metastasis	Operative approach	Recurrence rate, %	Survival, %
Konstantinidis et al. (2018) [1]	67		14.8	NA	LR or LT	NA	83.4 (5-year)
Lai et al. (2017) [2]	149	Percutaneous and/or surgical biopsy	NA	Lung, breast	LT and adjuvant therapy	24.8%	81 (5-year)
Dong et al. (2015) [3]	3	Liver biopsy	NA	NA	LR and RFA, or LT	0	100 (3-year)
Remiszewski et al. (2014) [4]	10	Liver biopsy during diagnostic laparoscopy	5.1	Lymph node metastases	LT	0	90 (5-year)
Noh et al. (2020) [5]	19	Liver biopsy	3.53	NA	LR, LR + chemotherapy, LT + radiation therapy, LT + chemotherapy	NA	88 (5-year)
Wang et al. (2018) [6]	1	Liver biopsy	4.7	No	LR + chemotherapy	0	100 (15-year)
Sanduzzi-Zamparelli et al. (2020) [7]	11	Surgical specimens, needle biopsy or “wedge-biopsy”		Lung, Lymph node metastases	LR or LT	36.4	100 (5-year)
Orlando et al. (2013) [8]	108	Percutaneous needle, surgical, or combined biopsies	NA	Osseous and peritoneal localizations	LT	NA	72 (5-year)
Jung et al. (2016) [9]	6	Liver biopsy	NA	NA	HR or LT	16.7	83.3 (5-year)
Abdoh et al. (2016) [10]	1	Liver biopsy	NA	Intrahepatic metastasis	LT	100	0 (1-year)
Theodosopoulos et al. (2013) [11]	5	Liver biopsy	4	Intrahepatic metastasis	Surgical resection with a non-formal hepatectomy or wedge resection.	40	60 (2-year)
Grotz et al. (2010) [12]	22	Liver biopsy	NA	Lung, peritoneum, bone, brain and skin	LR or LT	40	62 (LR, 5-year), 46 (LT, 5-year)
Wang et al. (2012) [13]	21	Surgical specimens	NA	Lung metastases, diaphragm/abdominal-wall metastases	LR, LR followed by TACE,or LT	NA	74.1 (LR, 3-year), 33.3 (LR followed by TACE, 3-year), 0 (LT, 3-year),
Thomas et al. (2012) [14]	7	Liver biopsy, diagnostic laparoscopy, surgical specimens	3.6	Lung	Hepatectomy or LT	43	83 (5-year)
Krasnodębski et al. (2020) [15]	18	Liver biopsy	NA	Hilar lymph nodes	LT	0	41.3 (5-year)
Thin et al. (2010) [16]	1	Liver biopsy	NA	Intrahepatic metastasis	LT	0	100 (5-year)
Lin et al. (2015) [17]	1	Surgical specimens	NA	No	LT	0	100 (5-year)
Samuk et al. (2016) [18]	1	Liver biopsy	NA	Lung	LT	0	100 (5-year)
Sundar et al. (2015) [19]	11	Liver biopsy	NA	NA	LT	NA	78.7 (5-year)
Groeschl et al. (2014) [20]	12	Liver biopsy, surgical specimens	NA	NA	Segmental resection, lobectomy/extended resection, LT	NA	57 (LR, 1-year), 80 (LT, 1-year),

*LR* liver resection, *LT* liver transplantation, *RFA* radiofrequency ablation, *HR* hepatic resection, *TACE* transcatheter arterial chemoembolization, *NA* not available

## Discussion

HEHE is an extremely rare borderline tumor of mesenchymal tissue-vascular endothelium origin and affects less than one in a million individuals [7]. The degree of malignancy of epithelioid hemangioendothelioma varies greatly between benign disease and aggressive cancer with widespread metastases. A case of meningeal epithelioid hemangioendothelioma with WWTR1-CAMTA1 gene fusion, growing slowly for more than 15 years, has been reported [8]. There have also been cases of patients with diffuse HEHE producing multiple splenic metastases and delayed multifocal bone metastases [9]. Primary HEHE refers to the invasion of the liver by a tumor originating in the liver mesenchyma rather than in other organs. HEHE consisting of a single lesion is considered to be the early stage of epithelioid hemangioendothelioma [3]. HEHE develops more frequently in males than in females, and appears to be associated with infections by hepatitis B- or C-viruses [10]. However, the exact pathogenesis of HEHE remains unclear.

The clinical presentation of most HEHE patients is nonspecific, with the pain in the right upper abdomen being the most common symptom. With the growth of volume and number of the masses, the occupied space increases, producing tension in the liver capsules and leading to pain. Additionally, the gradual enlargement of HEHE generates the risk of spontaneous rupture and hemorrhage [11]. HEHE has also been reported to cause secondary Budd-Chiari syndrome [12]. Other symptoms of HEHE include ascites, weight loss, jaundice, and liver enlargement. The most commonly reported biochemical abnormality associated with HEHE is the elevation of alkaline phosphatase, but the level of other liver enzymes, including γ-glutamyl transferase, lactate dehydrogenase, aspartate aminotransferase, and alanine aminotransferase, may also be increased.

All HEHE lesions showed low density on CT plain scan, and round areas of even lower density were detected within some lesions [13]. On MRI scans, HEHE lesions were characterized by nodules with low signal intensity on T1-weighted images and high signal intensity on T2-weighted images, compared to normal liver parenchyma. Other reported imaging features of HEHE include the “lollipop sign” and capsule retraction [13]. On contrast-enhanced CT and MRI, a variety of enhancements in the lesion were seen, including annular, delayed, and uneven enhancement; absence of enhancement was also present. Previous studies have documented that contrast-enhanced ultrasound of HEHE was characterized by an excessive rim or heterogeneous enhancement in the arterial phase, and marginal enhancement in the portal and late phases [14]. 18F-FDG PET/CT showed diffuse HEHE with mild to moderate hypermetabolism, multiple coalescent subcapsular hypodense lesions in the liver, and relative hypometabolism in the central area [15].

At present, the diagnosis of HEHE depends on the results of pathologic and immunohistochemical evaluation and is difficult to be obtained by an ultrasound-guided liver biopsy [16]. By immunohistochemistry, HEHE cells are positive for CD31, CD34, ERG, PCK, FLi-1, and TFE-3. Factor VIII-related antigen is expressed in almost 100% of HEHE, but the degree of its staining may be highly variable among the cells in the lesion [10]. The presence of CAMTA1-WWTR1 fusion products in HEHE helps to differentiate between hemangioma and angiosarcoma [17]. Typically, there is no clear association between the histology of liver lesions and the clinical course of the disease [10].

There is no standard treatment strategy for HEHE. A comprehensive literature review showed that the 1-year, 3-year, and 5-year overall survival rate of all 253 diagnosed individuals were 211 (83.4%), 141 (55.7%), and 104 (41.1%), respectively [18]. The most commonly used treatment for HEHE was hepatectomy, liver transplantation, radiotherapy and/or chemotherapy, and observational follow-up. Hepatectomy yielded the highest, up to 75%, 5-year survival rate [19]. Partial hepatectomy may not be a viable option for many patients due to high metastatic potential of HEHE, resulting in multiple tumor lesions. The reported chemotherapeutic agents include interferon-alpha, thalidomide, 5-fluorouracil, and monoclonal antibodies against vascular endothelial growth factor [19, 20]. Molecularly targeted drugs include sorafenib and apadinil [21]. Huaier granule is a water-based product of Huaier extract, which is approved traditional Chinese medicine by Chinese State Food and Drug Administration and can be used alone or in combination with other drugs to treat a variety of malignant tumors, including liver cancer [6]. Huaier granule exerts an anti-tumor response by inhibiting tumor angiogenesis and inducing cell cycle arrest at the G0/G1 checkpoint [22]. In addition, it regulates innate immunity by stimulating cytokine release and production of NO and reactive oxygen species [6]. A previous randomized, parallel controlled, nationwide multicenter study confirmed that the Huaier granule could reduce the recurrence of liver cancer after radical resection [6], and the risk of HEHE postoperative recurrence. However, the research on adjuvant therapy after the radical resection of HEHE only begins to develop.

Further studies designed as large multi-center trials would be needed to provide definitive guidelines for the diagnosis and treatment of HEHE. However, because HEHE is rare, the implementation of this type of studies presents a challenge. These circumstances highlight the value of reporting rare cases of HEHE. Although laparoscopic-guided liver biopsy has been used to verify HEHE diagnosis of HEHE [16], there are no previous reports of laparoscopic resection of HEHE. To the best of our knowledge, this is the first report of laparoscopic resection of hepatic epithelioid hemangioendothelioma.

## Conclusions

Primary HEHE is an extremely rare liver borderline tumor of vascular endothelial origin. This is the first report of laparoscopic hepatectomy of hepatic epithelioid hemangioendothelioma. Curative laparoscopic hepatectomy might be an acceptable treatment for appropriate HEHE patients.

## Supplementary information

**Additional file 1:.** References mentioned in Table 2

## Data Availability

The datasets used and/or analyzed during the current study are available from the corresponding author on reasonable request.
