# Supplementary material for: Laparoscopic resection of hepatic epithelioid hemangioendothelioma: report of eleven rare cases and literature review
Source: World J Surg Oncol. 2020 Oct 29;18:282. doi: 10.1186/s12957-020-02034-z (PMC7596953; doi:10.1186/s12957-020-02034-z)
Supplement: Supplementary file 1 — Additional file 1:. References mentioned in Table 2 [file 12957_2020_2034_MOESM1_ESM.docx]

**References mentioned in Table 2**

1. Konstantinidis IT, Nota C, Jutric Z, Ituarte P, Chow W, Chu P, et al. Primary liver sarcomas in the modern era: Resection or transplantation?. J Surg Oncol. 2018;117(5):886-91.

2. Lai Q, Feys E, Karam V, Adam R, Klempnauer J, Oliverius M, et al. Hepatic Epithelioid Hemangioendothelioma and Adult Liver Transplantation: Proposal for a Prognostic Score Based on the Analysis of the ELTR-ELITA Registry. Transplantation. 2017;101(3):555-64.

3. Dong K, Wang XX, Feng JL, Liu H, Zu KJ, Chang J, et al. Pathological characteristics of liver biopsies in eight patients with hepatic epithelioid hemangioendothelioma. Int J Clin Exp Pathol. 2015;8(9):11015-23.

4. Remiszewski P, Szczerba E, Kalinowski P, Gierej B, Dudek K, Grodzicki M, et al. Epithelioid hemangioendothelioma of the liver as a rare indication for liver transplantation. World J Gastroenterol. 2014;20(32):11333-9.

5. Noh OK, Kim SS, Yang MJ, Lim SG, Hwang JC, Cho HJ, et al. Treatment and prognosis of hepatic epithelioid hemangioendothelioma based on SEER data analysis from 1973 to 2014. HBPD INT. 2020;19(1):29-35.

6. Wang J-K, Wu Z-R, Su F, Ma W-J, Hu H-J, Li F-Y. Resectable Single Hepatic Epithelioid Hemangioendothelioma in the Left Lobe of the Liver: A Case Report. Open Med (Wars). 2018;13:456-9.

7. Sanduzzi-Zamparelli M, Rimola J, Montironi C, Nunes V, Alves VAF, Sapena V, et al. Hepatic epithelioid hemangioendothelioma: An international multicenter study. Dig Liver Dis. 2020;S1590-8658(20)30195-X.

8. Orlando G, Adam R, Mirza D, Soderdahl G, Porte RJ, Paul A, et al. Hepatic hemangiosarcoma: an absolute contraindication to liver transplantation--the European Liver Transplant Registry experience. Transplantation. 2013;95(6):872-7.

9. Jung D-H, Hwang S, Hong S-M, Kim K-H, Lee Y-J, Ahn C-S, et al. Clinicopathological Features and Prognosis of Hepatic Epithelioid Hemangioendothelioma After Liver Resection and Transplantation. Ann Transplant. 2016;21:784-90.

10. Abdoh QA, Alnajjar AM, Abaalkhail FA, Al Sebayel M, Al-Hussaini HF, Al-Hamoudi WK, et al. Aggressive Recurrence of Primary Hepatic Epithelioid Haemangioendothelioma after Liver Transplantation. Can J Gastroenterol Hepatol. 2016;2016:6135297.

11. Theodosopoulos T, Dellaportas D, Tsangkas A, Tsangkas N, Psychogiou V, Yiallourou A, et al. Clinicopathological features and management of hepatic vascular tumors. A 20-year experience in a Greek University Hospital. J BUON. 2013;18(4):1026-31.

12. Grotz TE, Nagorney D, Donohue J, Que F, Kendrick M, Farnell M, et al. Hepatic epithelioid haemangioendothelioma: is transplantation the only treatment option?. HPB (Oxford). 2010;12(8):546-53.

13. Wang L-R, Zhou J-M, Zhao Y-M, He H-W, Chai Z-T, Wang M, et al. Clinical experience with primary hepatic epithelioid hemangioendothelioma: retrospective study of 33 patients. World J Surg. 2012;36(11):2677-83.

14. Thomas RM, Aloia TA, Truty MJ, Tseng WH, Choi EA, Curley SA, et al. Treatment sequencing strategy for hepatic epithelioid haemangioendothelioma. HPB (Oxford). 2014;16(7):677-85.

15. Krasnodębski M, Grąt M, Morawski M, Wierzchowski M, Jastrzębski M, Remiszewski P, et al. Hepatic Epithelioid Hemangioendothelioma: A Rare Disease With Favorable Outcomes After Liver Transplantation. Transplant Proc. 2020;S0041-1345(20)30121-4.

16. Thin LWY, Wong DD, De Boer BW, Ferguson JM, Adams L, Macquillan G, et al. Hepatic epithelioid haemangioendothelioma: challenges in diagnosis and management. Intern Med J. 2010;40(10):710-5.

17. Lin Y-H, Lin C-C, Concejero AM, Yong C-C, Kuo F-Y, Wang C-C. Surgical experience of adult primary hepatic sarcomas. World J Surg Oncol. 2015;13:87.

18. Samuk I, Tekin A, Tryphonopoulos P, Pinto IG, Garcia J, Weppler D, et al. Abdominal transplantation for unresectable tumors in children: the zooming out principle. Pediatr Surg Int. 2016;32(4):337-46.

19. Sundar Alagusundaramoorthy S, Vilchez V, Zanni A, Sourianarayanane A, Maynard E, Shah M, et al. Role of transplantation in the treatment of benign solid tumors of the liver: a review of the United Network of Organ Sharing data set. JAMA Surg. 2015;150(4):337-42.

20. Groeschl RT, Miura JT, Oshima K, Gamblin TC, Turaga KK. Does histology predict outcome for malignant vascular tumors of the liver?. J Surg Oncol. 2014;109(5):483-6.
